# Regional Microglial Response in Entorhino–Hippocampal Slice Cultures to Schaffer Collateral Lesion and Metalloproteinases Modulation

**DOI:** 10.3390/ijms25042346

**Published:** 2024-02-16

**Authors:** Assunta Virtuoso, Christos Galanis, Maximilian Lenz, Michele Papa, Andreas Vlachos

**Affiliations:** 1 Neuronal Morphology Networks and Systems Biology Laboratory, Division of Human Anatomy, Department of Mental and Physical Health and Preventive Medicine, University of Campania Luigi Vanvitelli, 80138 Naples, Italy; 2 Department of Neuroanatomy, Institute of Anatomy and Cell Biology, Faculty of Medicine, University of Freiburg, 79104 Freiburg, Germany; christos.galanis@anat.uni-freiburg.de (C.G.); andreas.vlachos@anat.uni-freiburg.de (A.V.); 3 Hannover Medical School, Institute of Neuroanatomy and Cell Biology, 30625 Hannover, Germany; 4 Center for Basics in NeuroModulation (NeuroModulBasics), Faculty of Medicine, University of Freiburg, 79106 Freiburg, Germany; 5 Center BrainLinks–BrainTools, University of Freiburg, 79110 Freiburg, Germany

**Keywords:** microglia, astrocytes, brain injury, reactive gliosis, plasticity, neuroanatomy

## Abstract

Microglia and astrocytes are essential in sustaining physiological networks in the central nervous system, with their ability to remodel the extracellular matrix, being pivotal for synapse plasticity. Recent findings have challenged the traditional view of homogenous glial populations in the brain, uncovering morphological, functional, and molecular heterogeneity among glial cells. This diversity has significant implications for both physiological and pathological brain states. In the present study, we mechanically induced a Schaffer collateral lesion (SCL) in mouse entorhino–hippocampal slice cultures to investigate glial behavior, i.e., microglia and astrocytes, under metalloproteinases (MMPs) modulation in the lesioned area, CA3, and the denervated region, CA1. We observed distinct response patterns in the microglia and astrocytes 3 days after the lesion. Notably, GFAP-expressing astrocytes showed no immediate changes post-SCL. Microglia responses varied depending on their anatomical location, underscoring the complexity of the hippocampal neuroglial network post-injury. The MMPs inhibitor GM6001 did not affect microglial reactions in CA3, while increasing the number of Iba1-expressing cells in CA1, leading to a withdrawal of their primary branches. These findings highlight the importance of understanding glial regionalization following neural injury and MMPs modulation and pave the way for further research into glia-targeted therapeutic strategies for neurodegenerative disorders.

## 1. Introduction

Microglia, as integral components of the resident innate immune defense in the central nervous system (CNS), work alongside astrocytes in maintaining physiological networks. Their pivotal role is increasingly recognized in various neurological disorders [1]. Recent studies have challenged the traditional perspective of a homogenous glia population in the brain, revealing both morphological and molecular diversity among glial subpopulations, even in resting states [2,3]. The heterogeneity of glial morphology is mirrored by diverse functional capabilities [4], adding complexity to our understanding. This necessitates further investigation, particularly for the advancement of drug discovery and the development of personalized medicine approaches [5].

The phenotyping of astrocytes and microglia in CNS disease reveals distinct patterns based on their anatomical locations, as demonstrated through transcriptional, genetic, morphological, and metabolic activities [6,7]. Furthermore, the sensome of human microglia from various brain regions exhibits unique reactions to inflammatory stimuli, which change rapidly [8,9]. This introduces time as a critical variable in such studies, underscoring the dynamic nature of glial responses in the context of CNS disorders.

The activity of microglia and astrocytes, encompassing proliferation, motility, phagocytosis, and synaptic pruning, is influenced by the remodeling of the extracellular matrix (ECM), which is critical beyond the factors of origin, trigger, and timing of pathological processes [10]. Metalloproteinases (MMPs), zinc-dependent enzymes, are mainly responsible for the structural reorganization of the CNS during development, repair, and plasticity. The levels of MMPs are regulated by the components of the neurovascular unit, balancing between adaptive and maladaptive plasticity [11,12]. This regulation plays a significant role in determining the progression and severity of neurological disorders [13].

The modulation of MMPs has shown promising results in improving deficits in various disease models. In Alzheimer’s disease, MMPs modulation has led to notable improvements [14]. Similar beneficial effects have been observed in peripheral nerve injury [15,16] and in reducing tumor metastasis [17]. However, the impact of MMPs on hippocampal synaptic plasticity has presented inconsistent findings [18,19]. To understand the cellular and molecular mechanisms involving glial cell plasticity, various functional and mechanical lesion models have been established [20,21,22,23,24]. Despite these advancements, the precise links and underlying mechanisms connecting reactive gliosis, MMPs modulation, and the progression of neurological disorder remain to be fully elucidated.

In this study, we mechanically induced a Schaffer collateral lesion (SCL) in mouse entorhino–hippocampal slice cultures to transect the nerve fibers projecting to the pyramidal cells of cornu ammonis 1 (CA1) from cornu ammonis 3 (CA3). This approach was used to characterize microglia and astrocytes under MMPs modulation in the lesioned area, CA3, and the denervated region, CA1. The hippocampal subfields CA3 and CA1 share morphological and structural features including the laminar organization of neuro-glial cells and are often considered a continuum via the Schaffer collateral fibers [25,26]. However, CA3 and CA1 subserve specific, yet complementary functions, such as the encoding of memory acquisition, consolidation, and retrieval [27,28]. Indeed, the microdissection of both human and rodent hippocampal subfields contains differentially abundant proteins and responds selectively to modification of local cues in the environment as well as to ischemic and hypoxic conditions and neurodegeneration [29,30,31].

The cause of the hippocampal subfield heterogeneity may reside in a diverse susceptibility of the glial cells, the main modifiers of the neurovascular unit [10,32,33]. We analyzed adaptations of both microglia and astrocytes. Intriguingly, our findings revealed heterogenous responses of glial cells in CA3 and CA1. These results revealed the significant influence of anatomical location, the nature of the stimulus, and the timing of the disease process on glial cell behavior, and hence the complex dynamics of glial cell responses in CNS pathologies.

## 2. Results

### 2.1. Iba1+ Cells Respond to the SCL at 3 Days Post-Lesion (dpl), Unlike GFAP-Expressing Astrocytes

**Figure 1:**
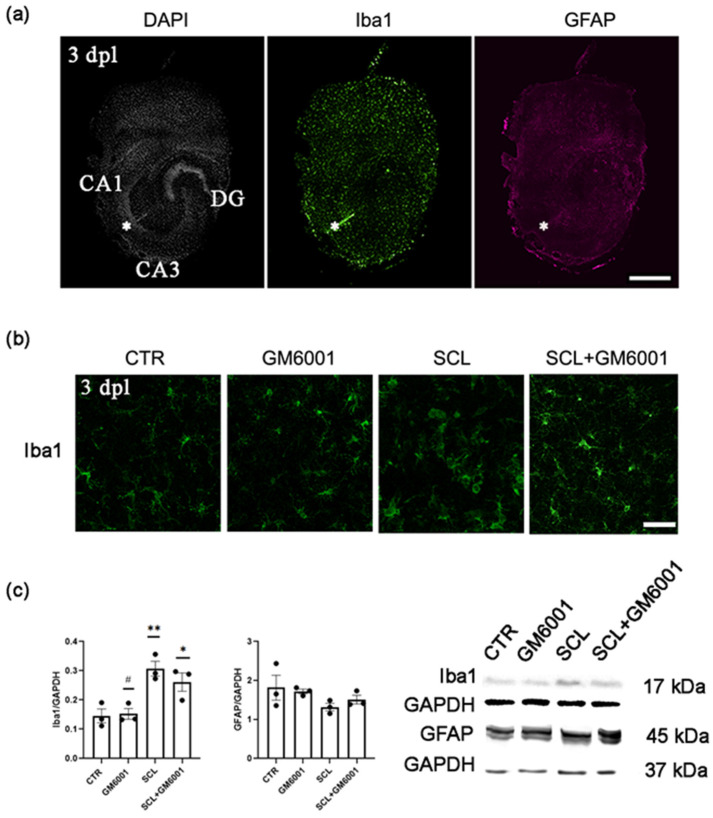
Microglia was activated three days after Schaffer collateral lesion (SCL) in vitro. (**a**) Representative entorhino–hippocampal slice culture stained for DAPI (white), Iba1 (green), and GFAP (magenta) 3 days after lesion (3 dpl). The SCL between hippocampal areas cornu ammonis 3 (CA3) and cornu ammonis 1 (CA1) is indicated with asterisks. Scale bar: 400 µm. (**b**) Representative image of Iba1-positive cells at the lesion site from CTR, GM6001, SCL, and SCL + GM6001 groups, 3 dpl. Scale bar: 50 µm. (**c**) Western blot relative quantification and corresponding bands for Iba1 (left) and GFAP (right) from whole control (CTR) and lesioned (SCL; 3 dpl) entorhino–hippocampal slice cultures. A set of slice cultures was treated with the broad-spectrum MMPs inhibitor GM6001 (50 nM; *n* = 3 cultures per group). (*) was used to show the significant differences relative to the CTR group, while (#) was used when detected through multiple comparisons among the experimental groups. (#) = *p* < 0.05; (*) = *p* < 0.05; (**) = *p* < 0.01.

Three days after the SCL, a notable expression of Iba1-positive cells, indicative of microglia, was observed around the lesion site in the entorhino–hippocampal slice cultures. Conversely, the immunostaining for GFAP, a marker for astrocyte activation, was not noticeable at the lesion site (Figure 1a), implying a differential glial response to the injury.

Further examination using a high-magnification lens confirmed the increase in Iba1-responsive elements at the lesion site. Interestingly, treatment with GM6001 ameliorated the microglial reaction (Figure 1b).

Western blot analysis conducted on the whole slices revealed a significant increase in Iba1 expression in the SCL group, while GFAP levels did not show any significant differences among the experimental groups (Figure 1c and Figure S1). Notably, Iba1 overexpression was modestly reduced following GM6001 [50 nM] treatment, although this decrease was not statistically significant (Figure 1c and Figure S1).

These data suggested that SCL selectively triggered a microglial response at early stages post-injury, without significantly impacting the reactivity of GFAP-expressing astrocytes at 3 days post-lesion (dpl).

### 2.2. Iba1+ Cells Show a Region-Specific Response to the SCL in Entorhino–Hippocampal Slice Cultures

**Figure 2:**
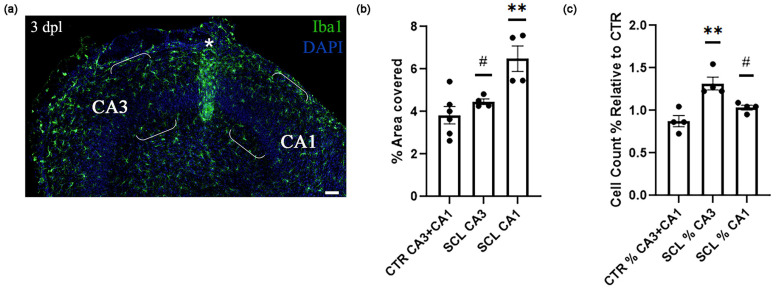
Region-specific effects of SCL on microglia. (**a**) Hippocampal slice culture stained for Iba1 (green) and DAPI (blue), 3 dpl. Microglia were analyzed in specified regions of CA3 and CA1, 0.5 mm lateral to the lesion site (marked with an asterisk). Scale bar: 200 µm. (**b**) Data analysis of percentage area covered by Iba1-expressing cells relative to the total scanned area (*n* = four–six cultures per group, 3 dpl). (**c**) Iba1-positive cell count relative to the cell count in the pooled CTR group (%) (*n* = four cultures per group, 3 dpl). (*) was used to show the significant differences relative to the CTR group, while (#) was used when detected through multiple comparisons among the experimental groups. (#) = *p* < 0.05; (**) = *p* < 0.01.

Iba1 protein overexpression is a key feature of reactive gliosis [34], potentially resulting from cell hypertrophy and/or increased microglial density. To investigate this, hypertrophy measurements and cell counts were conducted in a representative area of CA3, located 0.5 mm from the lesion site, and in a symmetrical region in CA1, as depicted in Figure 2a. Iba1 immunostaining effectively highlights both cell bodies and processes, facilitating the assessment of morphological diversity among myeloid cells [35]. This made Iba1 a valuable marker for quantifying the extent of microglial activation, allowing for both the measurement of the area occupied by Iba1+ cells, serving as a hypertrophy index, and cell counting, as shown in Figure 2b,c.

The analysis revealed a notable difference in the response of Iba1+-expressing cells across regions. Specifically, the area covered by Iba1+ cells in CA1 was significantly larger compared to that in both the control (CTR) group and CA3 (Figure 2b). Interestingly, this increase in coverage did not correspond to a significant increase in the cell count in CA1, while it was supported by the maintenance of the primary process length compared to the CTR (Table 1). In contrast, the microglia in CA3 did not exhibit hypertrophy when compared to those in the CTR group, yet there was an observable increase in number (Figure 2c, Table 1).

Taken together, these data highlight a distinct regionalization in the response of Iba1-expressing cells to the SCL. The regional variation pointed to a nuanced heterogeneity, possibly attributed to the differential nature of the stimulus: direct nerve damage in CA3 and denervation to CA1. This suggested that the microglial response was finely tuned not only to the presence of injury but also to the specific type of neural insult.

### 2.3. MMP Modulation Leads to a Reorganization of the CA1 Microglial Response to SCL

Microglial activities such as proliferation, hypertrophy, and motility within the neuro-glial network require precise remodeling of the ECM, largely driven by MMPs activity [13]. Notably, MMPs are implicated in hippocampal synaptic plasticity in both the CA3 and CA1 regions, although the underlying molecular mechanisms may significantly differ [36]. Furthermore, CA1 and CA3 exhibit notable differences in their transcriptional and proteomic profiles under basal conditions [30,31].

**Figure 3:**
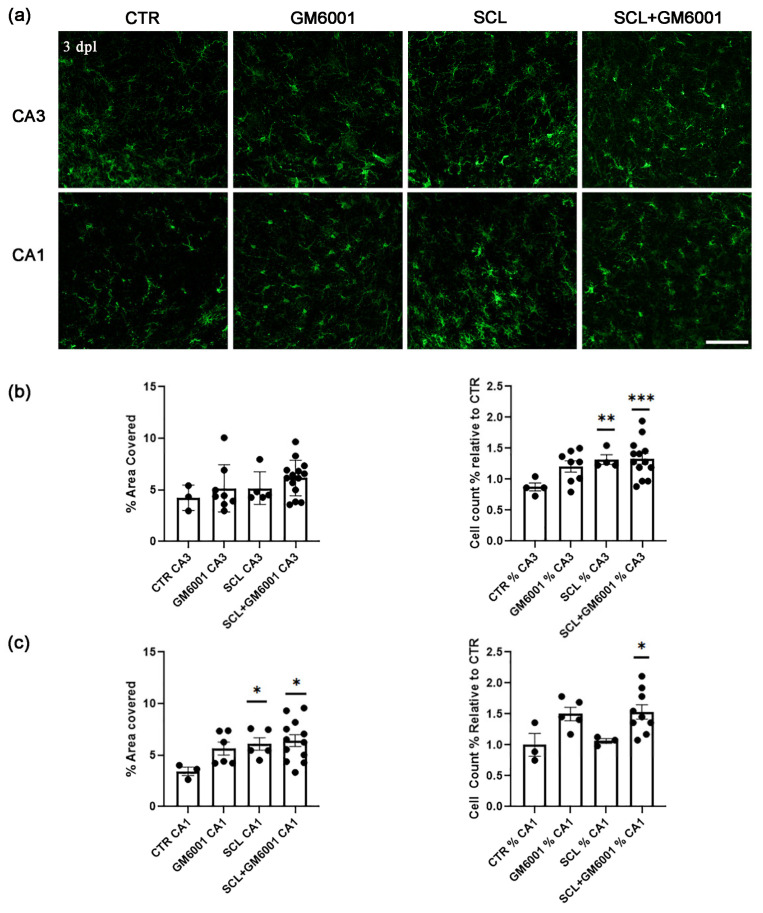
Effects of metalloproteinases on SCL-induced microglia responses. (**a**) Representative images of Iba1-positive cells (green) from CTR, GM6001, SCL, and SCL + GM6001 groups, 3 dpl. Scale bar: 100 µm. (**b**,**c**) Analysis of percentage area covered by Iba1-expressing cells relative to the total scanned area (left), and cell count expressed in percentage relative to the CTR (right) (CA3, *n* = 4–13 cultures per group; CA1, *n* = 3–12 cultures per group; 3 dpl). (*) was used to show the significant differences relative to the CTR group. (*) = *p* < 0.05; (**) = *p* < 0.01; (***) = *p* < 0.001.

In this context, treatment with the MMPs broad spectrum inhibitor GM6001 [50 nM] for 3 days did not affect the microglial coverage area and count in CA3 under control conditions and 3 dpl (Figure 3a,b). However, the treatment with GM6001 induced a slight withdrawal of the primary processes, which were reshaped with the SCL (Table 1). In contrast, with GM6001 treatment, CA1 microglia maintained hypertrophic characteristics associated with SCL and exhibited an increased cell number and a reduction in the primary branch length in comparison with those in the CTR group (Figure 3a–c; Table 1).

These results supported the concept of region-specific microglial responses following MMPs modulation, emphasizing the complexity and diversity of glial plasticity. They highlighted the importance of a detailed understanding of regional and molecular variations in glial cell responses within the CNS.

## 3. Discussion

CNS plasticity has been a subject of extensive study, with the hippocampus often serving as a primary model due to its distinct structure. Encased by archicortical and neocortical structures, the hippocampus can mimic a “closed” network of a higher order, providing a unique environment for studying neural dynamics and plasticity. Entorhino–hippocampal slice cultures are particularly valuable in this context as they allow for in vitro investigation while preserving the complex cyto- and fiber architecture of the neuroglial network. This makes them an effective experimental model for studying the morphological, molecular, and functional dynamics of glial cells [22,37,38].

Glial cells are increasingly recognized for their pivotal role in various neurological disorders, including brain trauma, spinal cord injury, and neurodegenerative diseases [3,39]. Yet, a comprehensive understanding of glial plasticity, encompassing the nature of the trigger, the timing of the reactive gliosis, and the specificity of the brain region involved, remains an area in need of further research [40].

In this study, we utilized entorhino–hippocampal slice cultures and mechanically induced a lesion to transect Schaffer collaterals connecting CA3 to CA1. Three days after SCL, there was a marked increase in Iba1 protein expression in the hippocampal lysate, indicating an early microglial response, with no simultaneous alterations in GFAP levels, suggesting that astrocytes did not initially activate to the same extent. Accordingly, Iba1-expressing microglia at the lesion site appeared to be activated with an ameboid shape, typical for engulfing debris, releasing cytotoxins, and secreting astroglial growth factors [41]. This suggested that microglia are swift to detect lesion-induced changes within the neuroglial network and may act as initial responders in reactive gliosis [42,43].

Microglial cells may trigger neuroinflammation, promoting astrocyte activation, while GFAP-expressing astrocytes appear to significantly increase their activity at a later time [42,44]. However, the effects of microglia on astrocytes may vary across functional states. Microglia are thought to modify the synaptic structure and neuronal activity through an interplay with astrocytes by controlling the expression of connexins, glutamatergic synapse density, and expression of post-synaptic scaffold proteins in CA1 [45].

Our experiments further highlighted the regional specificity of the microglial responses to SCL in CA3 and CA1. In CA3, microglia showed an increase in density, suggesting that cell migration and/or proliferation occurred as rapid responses to direct neural injury [46]. In contrast, CA1 microglial cells exhibited hypertrophy, a feature of activated microglia characterized by enlarged somata and processes, and sometimes a reduction in ramification [47]. Hypertrophied microglia appear to be distinct from ameboid, phagocytic fully activated microglia, which are enlarged and have a round body with minimal or no processes [48]. Beyond the morphology, multiple states of microglial activation imply a diverse cytoskeleton, migration ability, the production and usage of ECM-degrading enzymes, as well as MMPs endogen inhibitors [32].

The denervation of CA1 neurons may require highly coordinated modifications in the neuroglial network compared to direct damage to CA3 axons, especially considering that (i) CA1 dendritic spine density drops early after the lesion [49,50], (ii) dendrites retract [51], and (iii) the transected CA3 axons degenerate and are cleared by glial cells in the denervated region [52,53].

The proliferation, migration, and activation of microglia in the CA3 region seemed not to depend on MMPs-mediated ECM remodeling during the early phase after SCL, since the microglial morphology and counts were unaffected by the GM6001-induced modulation of MMPs. Previous research on spinal cord networks has revealed elevated MMPs levels following peripheral nerve injury [54,55]. Additionally, it has been found that reactive gliosis can be reduced by inhibiting MMPs at later stages after injury [16,56]. Hence, the MMPs-dependent remodeling of ECM in CA3 may account for changes seen in later stages, such as postlesional axonal sprouting [57].

In CA1, administering GM6001 resulted in an increased microglial density and a smaller length of primary branches while preserving hypertrophy. CNS microglial repopulation is characterized by an increase in cell density along with a reduction in ramification length [58]. The withdrawal of microglial processes may correspond to a decrease in the surveillance function [59] in favor of compelling activities, suggesting that MMPs influence microglial responses in denervated brain regions during the early post-lesion phase.

The biological significance of these alterations is yet to be determined. Earlier research has shown denervation-induced spine density changes in CA1, but no significant functional alterations in CA1 neurons post-SCL after 3 days [22]. The transcriptomic analysis in this previous study also revealed higher MMP16 mRNA levels post-SCL [22], a membrane-bound MMP expressed by microglia, potentially involved in synaptic rewiring and hippocampal spine density regulation [22,60,61]. Regardless of these considerations, the results of the present study clearly showed distinct responses in glial cells following SCL and with GM6001, a broad-spectrum MMPs inhibitor.

Microglial regionalization to injury and treatments may provide localized homeostatic functions and could underlie region-specific sensitivities to dysregulation-related neurodegenerative diseases. It is known that neurodegeneration occurs in areas that appear to be disease-specific. However, its origin is unknown [62,63,64]. Local differences in the microenvironment, such as matrix composition, blood–brain barrier permeability, neurotransmitter profiles, heterogeneity in other cell types, epigenetic landscapes of gene expression, and timing and nature of the stimuli may all be important to determine microglial diversity [64,65,66].

Understanding the genetic and neurobiological mechanisms that underlie these differences will be essential in further studies. Using spatial single-cell technology, a recent investigation showed that mouse hippocampal CA3 and CA1 subregions did not include a single transcriptional profile per cell class. Instead, distinct combinations of cell expression clusters were found [67]. The subregional morphological features of CA3 and CA1 microglia may correspond to several transcriptional profiles, going beyond the simplistic classification of activated, pro-inflammatory (M1) and anti-inflammatory (M2) states [68,69].

Accordingly, the majority of microglial genes associated with classical (M1) or alternative (M2) activation, including the marker genes *iNos* and *Arg1*, respectively, was expressed at almost undetectable levels in 4-month-old mouse brains. This suggested that hippocampal microglia are distinct from conventional states of activation [64]. Additionally, in controlled, healthy conditions, mouse microglia is characterized by hyper-ramification and homeostatic transcriptional signatures related to the immune-alert state, which is overrepresented in the hippocampus compared to that in other brain regions. The immune–surveillance state of microglia is associated with energy metabolism [70], suggesting that the metabolic environment of microglia could drive the region-dependent regulation of gene expression. Indeed, steady-state differences in microglial genotypes could also contribute to region-dependent functions [64].

## 4. Materials and Methods

### 4.1. Ethics Statement

Mice were maintained in a 12 h light/dark cycle with food and water available ad libitum. All experimental procedures were performed according to the German animal welfare legislation and approved by the animal welfare committee and/or the animal welfare officer at the University of Freiburg, Faculty of Medicine (X-17/07K, X-18/02C, X-21/01B). All the procedures were conducted with care to minimize distress and pain to the animals.

### 4.2. Preparation of Entorhino–Hippocampal Organotypic Tissue Cultures

Entorhino–hippocampal tissue cultures were prepared at postnatal day 4–5 from C57BL/6J mice of either sex as previously described [37]. Slices 300 μm in length were cut using the vibratome, maintaining the sterility. The cultivation medium contained 50% (*v*/*v*) MEM, 25% (*v*/*v*) basal medium eagle, 25% (*v*/*v*) heat-inactivated normal horse serum, 25 mM HEPES buffer solution, 0.15% (*w*/*v*) bicarbonate, 0.65% (*w*/*v*) glucose, 0.1 mgmL^−1^ streptomycin, 100 UmL^−1^ penicillin, and 2 mM glutamax. The pH was adjusted to 7.3 and the medium was replaced three times per week. All tissue cultures were allowed to mature for at least 18 days to ensure the stability of the structural and functional properties of the organotypic tissue [23,71,72,73]. Entorhino–hippocampal tissue cultures were maintained in a humidified atmosphere with 5% CO_2_ at 35 °C.

### 4.3. Schaffer Collateral Lesion (SCL)

Mechanical pathway transection was performed with a sterile scalpel in mature tissue cultures (≥18 days in vitro) as described in [22]. SCL was applied between the CA3 and the CA1 region of the hippocampus, without affecting the perforant path projections to CA3 (Figure 1a). Except for the lesion-induced partial denervation of CA1 pyramidal neurons, the cytoarchitecture of both the hippocampus and the entorhinal cortex remained unchanged (Figure 1a).

### 4.4. Pharmaceutical Treatment for MMPs Modulation

MMPs modulation was performed using a broad-spectrum MMP inhibitor, GM6001, Ilomastat (Merck, Millipore, Burlington, MA, USA). GM6001 (Ilomastat, Galardin) is a nonspecific hydroxamic acid-based dipeptide with potent inhibitory activity against collagenases (MMP1, MMP8, and MMP13), gelatinases (MMP2 and MMP9), and stromelysin (MMP3, MMP10, and MMP11) among MMPs [74]. GM6001 was prepared as per manufacturer instructions and dissolved in dimethyl sulfoxide (DMSO). GM6001 was then added in the slice culture medium at a final concentration of 50 nM as a pretreatment, 1 h before the SCL. Fresh GM6001 was added every time the medium was replaced [75].

### 4.5. Experimental Groups

Slice cultures were divided into four groups: (I) control, receiving 1 μL of DMSO in the fresh cultivation medium; (II) GM6001, having 1 μL of GM6001 50 μM in the fresh cultivation medium; (III) SCL, subjected to the Schaffer collateral lesion (SCL); and (IV) SCL + GM6001, receiving the lesion after 1 h of pre-treatment with GM6001.

### 4.6. Immunofluorescence (IF)

At 3 dpl, the slices were processed for immunostaining. Cultures were fixed in a solution of 4% (*w*/*v*) paraformaldehyde (PFA) in phosphate-buffered saline (PBS, 0.1 M, pH 7.4) and 4% (*w*/*v*) sucrose for 1 h. Fixed cultures were incubated for 1 h with 10% (*v*/*v*) normal goat serum (NGS) in 0.5% (*v*/*v*) Triton X-100-containing PBS to block non-specific staining. Whole tissue cultures were incubated with rabbit anti-Iba1 (1:1000; Fujifilm Wako, Richmond, VA, USA, #019–19741) or mouse anti-GFAP (1:1000; Sigma, St. Louis, MO, USA) in PBS containing 10% (*v*/*v*) normal goat serum (NGS) and 0.1% (*v*/*v*) Triton X-100 at 4 °C overnight. Cultures were washed and incubated for 3 h with appropriate secondary antibodies (Alexa Fluor anti-rabbit 488; Alexa Fluor anti-mouse 555, 1:1000, in PBS with 10% NGS or NHS, 0.1% Triton X-100; Invitrogen, Waltham, MA, USA). All the nuclei were visualized by DAPI staining (#62248, 1:5000 in PBS for 10 min; Thermo Scientific, Waltham, MA, USA). Sections were washed, transferred onto glass slides and mounted for visualization with an anti-fading mounting medium (DAKO Fluoromount, Santa Clara, CA, USA).

Confocal images of the immunostainings were acquired using a Leica SP8 confocal microscope equipped with a 20× (NA 0.75, Leica) or 40× (NA 1.3, Leica) objective lens. The detector gain and amplifier were initially set to obtain pixel intensities within a linear range.

### 4.7. Western Blotting (WB)

At 3 dpl, the slices were processed for Western blotting. Whole entorhino–hippocampal slice cultures were homogenized in RIPA buffer enriched with phenylmethanesulfonyl fluoride (PMSF) and a protease inhibitor cocktail. The protein content of each sample was determined using the Bradford method and the same amount of proteins was loaded in polyacrylamide gels (10–15%). The proteins were separated during the SDS page and transferred to a PVDF membrane using the Biorad Tetra System. After blocking in 5% non-fat milk in T-TBS (Tris buffer phosphate + Tween) for 35 min, the membranes were incubated with the primary antibody against GFAP (mouse, 1:1000, Sigma, St. Louis, MO, USA), Iba1 (rabbit, 1:500, Wako, Richmond, VA, USA, #019–19741), and GAPDH (rabbit, 1:500, Novus-Bio, Easter Ave Centennial, CO, USA ) at 4 °C. After incubation with the proper secondary antibody, the detection of the targets was performed using chemiluminescence. The expression of the Iba1 and GFAP band was normalized to the expression of the corresponding loading control, GAPDH. WB experiments were repeated at least three times.

### 4.8. Quantification and Statistics

All the quantifications were performed using Image J 2.0 software. A semi-automated procedure was conducted to quantify non-laminar microglial hypertrophy and the cell count in the CA1 and CA3 regions. Hypertrophied microglia are characterized by an enlarged somata, occasionally with shorter, thicker, and less branched processes, distinct from ameboid, phagocytic fully activated microglia [47,76]. The hypertrophy index was assessed as the percentage (%) of area covered by the Iba1+ cells compared to the total scanned area in CA1 and CA3 using a fixed ROI (region of interest) spanning from the stratum oriens to stratum moleculare. The primary branch length from the soma was measured for at least four cells in each ROI using the tubeness plugin from Image J 2.0 Software. The tubeness filter allowed to score how “tube-like” each point was, facilitating the visualization and length measurement of the microglial primary ramifications. The Iba1+ cell count was expressed as percentage relative to the number of cells identified in CA3 and CA1 of the CTR group, respectively. Immunostaining files were renamed using a code, and the analysis was conducted blinded.

Data were statistically analyzed using GraphPad Prism 9 (GraphPad software, USA). Normal (Gaussian) distribution was tested using the Shapiro–Wilk test. For the statistical comparison of data sets with three or more experimental groups passing the normality test, a one-way ANOVA test with Tukey’s correction for multiple comparisons was used. For the evaluation of data sets with three or more experimental groups not distributed under a Gaussian curve, the Kruskal–Wallis test followed by Dunn’s post hoc correction was applied. *p*-values < 0.05 were considered to be statistically significant (* *p* < 0.05, ** *p* < 0.01, *** *p* < 0.001). The n-numbers are provided in the figure legends. (*) was used to show the significant differences relative to the CTR group, while (#) was used when detected through multiple comparisons among the experimental groups. In the text and figures, data are expressed as mean ± standard error of the mean (s.e.m.).

### 4.9. Digital Illustrations

Confocal image stacks were stored as TIF-files. Figures were prepared using the Image J 2.0 software and Adobe Photoshop CC2017 graphics software. Images were adjusted for brightness, contrast, and sharpness, maintaining the same parameters among the groups.

## 5. Conclusions

The present study highlighted the significance of region-specific localization in the CNS with respect to glial cell behavior. It showed that glial responses can differ based on the specific CNS region involved, the nature of the initiating event, and the timing of neural plasticity. Enhancing our understanding of the region-specific (and time-dependent) dynamics of glial cells in pathological states would certainly provide deeper insights into both adaptive and maladaptive processes. Such knowledge is crucial for developing targeted glia-specific therapeutic strategies for neurodegenerative disorders.

## Figures and Tables

**Table 1:** Region-specific effects on primary process length of microglia, 3 dpl. Hypertrophied microglia may show a reduction in the process length. Data are expressed as the mean length (μm) per primary process ± s.e.m. from the microglial soma. (*) was used to show the significant differences relative to the CTR group, while (#) was used when detected through multiple comparisons among the experimental groups. (*) = *p* < 0.05; (**) = *p* < 0.01; (***) = *p* < 0.001. (#) = *p* < 0.05; (###) = *p* < 0.001.

	CTR	GM6001	SCL	SCL + GM6001
CA3	24.77 ± 2.171	16.46 ± 0.8683 *	20.99 ± 1.385	19.53 ± 0.9217
CA1	29.86 ± 2.766	18.11 ± 1.076 ** ^#^	22.63 ± 1.347	15.38 ± 0.6072 *** ^###^

## Data Availability

Data is contained within the article and Supplementary Materials.

## Supplementary Materials

The following supporting information can be downloaded at: https://www.mdpi.com/article/10.3390/ijms25042346/s1.
