# Supplementary material for: Regional Microglial Response in Entorhino–Hippocampal Slice Cultures to Schaffer Collateral Lesion and Metalloproteinases Modulation
Source: Int J Mol Sci. 2024 Feb 16;25(4):2346. doi: 10.3390/ijms25042346 (PMC10889226; doi:10.3390/ijms25042346)

Figure S1

Representative original blots for Iba1, and their corresponding internal control GAPDH.

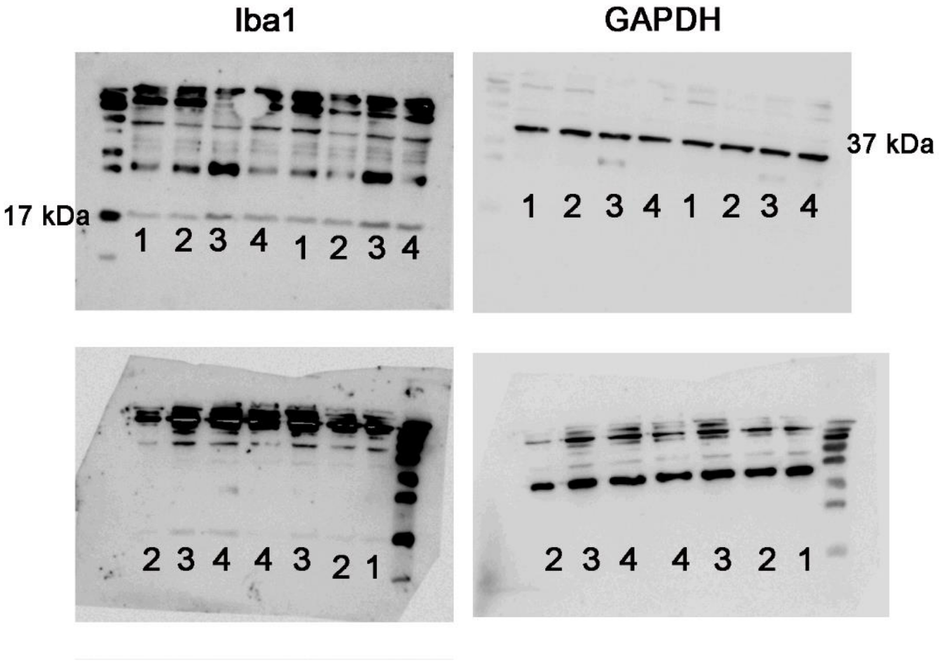

Representative original blots for GFAP, and their corresponding internal control GAPDH.

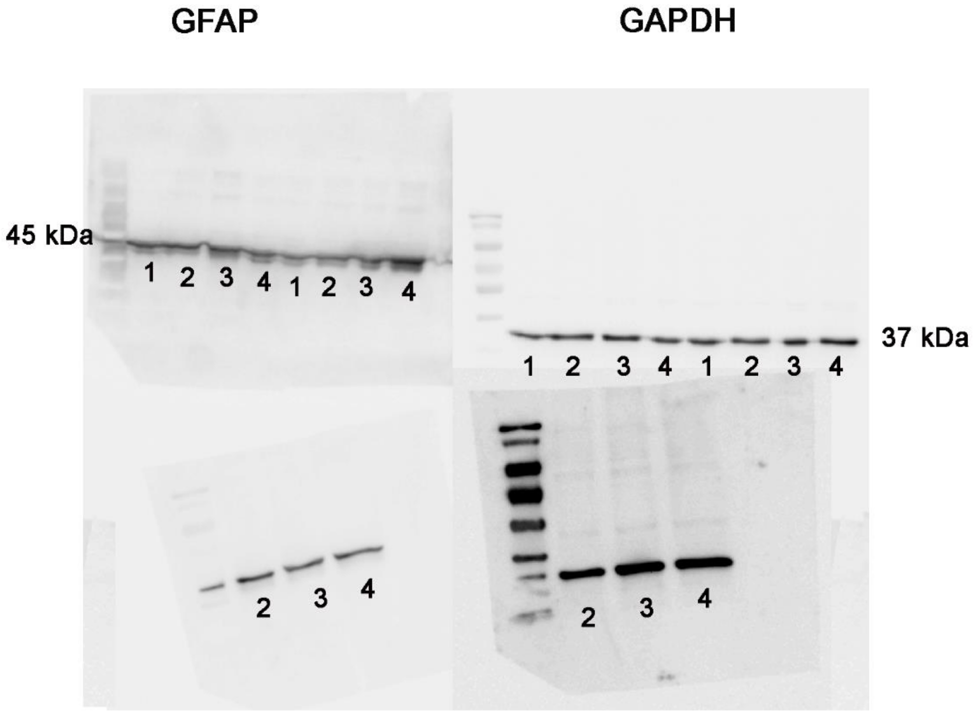

Supplement: Supplementary file 1 [file ijms-25-02346-s001.zip › ijms-2845901-supplementary.pdf]
